# Predicting Success of Two-Stage Exchange for Prosthetic Joint Infection Using C-Reactive Protein/Albumin Ratio

**DOI:** 10.1155/2019/6521941

**Published:** 2019-05-02

**Authors:** Cierra S. Hong, Sean P. Ryan, Jonathan A. Gabor, Michael A. Bergen, Ran Schwarzkopf, Thorsten M. Seyler

**Affiliations:** ^1^Duke University Medical Center, Durham, NC, USA; ^2^NYU Langone Health, New York City, NY, USA

## Abstract

Two-stage exchange is most commonly used for treatment of prosthetic joint infections (PJI) but, this may fail to eradicate infections. C-reactive protein/albumin ratio (CAR) has been used to predict survival and operative success in other surgical subspecialties and so, we assess the association between CAR and reimplantation success during two-stage revision for PJI defined by the Musculoskeletal Infection Society following a primary total hip (THA) or knee (TKA) arthroplasty. From January, 2005 to December, 2015, two institutional databases were queried and patient demographics, antibiotic duration, C-reactive protein, and albumin were collected prior to reimplantation. Two-stage revisions were considered successful if patients were off of antibiotics and did not require a repeat surgery. CAR was available for 79 patients (34 hips and 46 knees) with 61 successful two-stage revisions and 18 failures. The average CAR for patients with successful reimplantation was 1.2 (0.2, 3.0) compared to 1.0 (0.4, 3.2) for treatment failure. However, this was not statistically significant (p=0.766). Therefore, CAR is not applicable in predicting the prognosis of two-stage revisions for PJI in total arthroplasty but other preoperative inflammatory-based prognostic scores should be explored.

## 1. Introduction

In the United States, total hip arthroplasty (THA) and total knee arthroplasty (TKA) are two of the most commonly performed surgeries that treat arthritic pain and improve a patient's functional status. The number of procedures is projected to grow to about half a million and 1.5 million by 2020, respectively [1]. However, this also increases the risk for postoperative complications, including prosthetic joint infections (PJIs), an uncommon but expensive and debilitating complication. From 2001 to 2009, the incidence of PJIs annually in THA and TKA increased from about 2% to 2.18%, almost a twofold increase in the number of infected cases, which led to an additional $200 million in inpatient care costs in the United States [2].

There are multiple strategies to treat and minimize PJI and its associated morbidity and mortality. They include the following medical and surgical managements: debridement, antibiotic, and implant retention (DAIR); one-stage arthroplasty exchange (resection prosthesis followed by reimplantation at the time of explant); two-stage arthroplasty exchange (resection prosthesis followed by reimplantation at a later time point from the explant); and resection arthroplasty [3, 4]. Arthrodesis, amputation, and/or antimicrobial suppression are reserved for chronic or resistant infections [5–7]. In the United States, two-stage exchange is the preferred procedure for eradicating PJIs due to the strong literature support which shows successful outcomes in THA [8] and TKA [9, 10]. However, despite at least a 70% infection-free survival over 10 years [9, 11], those who are not successfully treated with a two-stage revision arthroplasty experience severe complications that can result in soft tissue deficiencies, arthrodesis, amputations, or death [12]. This led to studies investigating risk factors, such as polymicrobial infections and multiple revision surgeries prior to explant of the primary arthroplasty, that predict two-stage revision failures in THA and TKA in order to optimize surgical and medical management of these patients [13, 14].

In other surgical specialties, novel preoperative systemic-inflammation based prognostic scores evaluating C-reactive protein (CRP) and albumin have been shown to predict surgical and overall survival outcomes in addition to risk for disease recurrence after oncologic resections [15–17]. Specifically, the CRP albumin ratio (CAR) has been shown to successfully predict prognosis in many types of cancers most likely because it combines a hallmark of tumorigenesis, inflammation, and preoperative nutritional status [18–21]. However, to our knowledge, CAR has not been investigated in assessing two-stage reimplantation outcomes for PJI in total arthroplasty. In this study, we aim to [1] report the preoperative CAR from patients undergoing a two-stage reimplantation for PJI from two different institutions and [2] assess the correlation with successful postoperative outcomes. We hypothesized that patients who had a successful treatment of their PJI after a two-stage revision for THA or TKA will have elevated preoperative CAR values.

## 2. Materials and Methods

From January 2005 to December 2015, two institutional databases at tertiary referral centers were retrospectively queried for patients who had a two-stage exchange for PJI after a primary THA or TKA. Patients were included for analysis only if they (1) met either one of the two major or four of the six minor criteria for PJI as defined by the Musculoskeletal Infection Society (MSIS) [22]; (2) completed an explant of their primary THA or TKA and reimplantation at a different surgery date; and (3) had a serum CRP and serum albumin value that were collected within one month of each other and were completed no later than one year before their explant. A total of 79 patients met these inclusion criteria and were retrospectively enrolled in the study. One patient had a two-stage revision for both a primary THA and TKA.

In addition to laboratory data including serum CRP and albumin, clinical demographics, such as age and gender, and antibiotic management were collected. A two-stage exchange arthroplasty was classified successful based off an international multidisciplinary consensus defining successful PJI eradication published by Diaz-Ledezma et al. known as the Delphi consensus criteria [23]. The consensus included the following: (1) no additional surgery to the affected joint after reimplantation; (2) complete PJI eradication which includes no life-long antibiotics for antimicrobial suppression or reinfection by the same organism; and (3) no causes of death related to PJI (i.e., sepsis).

Statistical analysis was performed using Wizard Pro for Mac (E. Miller, Chicago, IL). A univariable logistic regression analysis was performed to assess the primary endpoint, which was whether the two-stage exchange was successfully treating PJI at the two institutions included in the study. Continuous data are presented as median (lower quartile; upper quartile) and were analyzed with a Mann Whitney test between the two cohorts (successful versus unsuccessful two-stage revision) while categorical data are presented as count (percent) and were analyzed with chi-squared test. The results were considered statistically significant when p<0.05.

## 3. Results

**Figure 1:**
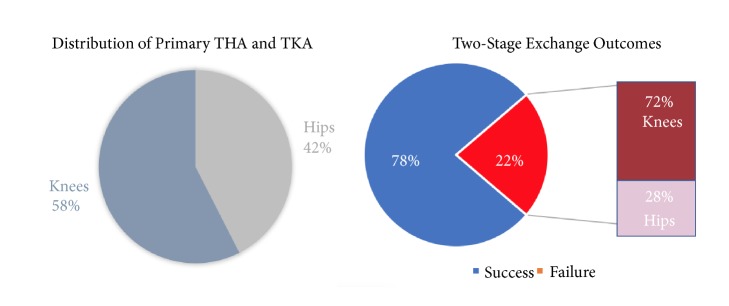
*Left: *distribution of patients included in study cohort.* Right, *distribution of two-stage exchange outcomes after primary THA and TKA.

From two institutional databases, a total of 79 patients (34 primary THA; 46 primary TKA) developed a PJI as defined by the MSIS and completed a two-stage exchange from 2005 to 2015. Sixty-one of these revisions successfully treated the patient's PJI whereas 18 total joint arthroplasty (5 THA; 13 TKA) required additional surgeries and/or chronic antimicrobial suppression (Figure 1). Demographic data, such as age and gender, in addition to laboratory values of interest are listed in Table 1 for these patients.

Between the two cohorts treated with two-stage exchange for PJI, the median CRP (p=0.713) and albumin (p=0.263) were not significantly different. Additionally, the preoperative CAR was not statistically significant between the cohort with a successful two-stage reimplantation compared to those with a failed reimplantation (successful CAR 1.2 (0.2, 3.0) versus failed CAR 1.0 (0.4, 3.2); p=0.766). In the univariable logistic regression analysis, when considered in isolation, neither CRP (OR 1.00, 95% CI 0.97-1.02; p=0.671) nor albumin (OR 0.99, 95% CI 0.09-1.05; p=0.946) adequately predicted failure of revision. Similarly, CAR did not predict failure (OR 0.975, 95% CI 0.90-1.06; p=0.561).

Forty-two patients (53.2%) had CAR obtained within 30 days of surgery. Thirty of these patients had successful reimplantation and 12 had two-stage exchange revision failures with a CAR median of 1.20 (0.19, 3.43) and 1.03 (0.45, 2.38), respectively. There was no significant difference in preoperative CAR between these two groups (p=0.945) (Table 2).

## 4. Discussion

PJI, also known as periprosthetic joint infection, can compromise the prosthesis and surrounding bone and soft tissue structures, which significantly increases a patient's morbidity and can lead to patient mortality [4, 24]. In 2011, the MSIS created the major and minor criteria in order to standardize the definition of PJI. It consists of periprosthetic cultures, elevated lab values including C-reactive protein (CRP) and erythrocyte sedimentation rate (ESR), and the presence of a sinus tract into the affected joint [22]. As previously mentioned, in the United States, in addition to antibiotic management, PJI is commonly managed with two-stage exchange arthroplasty due to its success with infection eradication and postoperative survival. However, the outcomes are variable with a reported failure rate up to a third of two-stage knee reimplantations [25] and a tenth of two-stage hip reimplantations [3]. Risk factors including the type of organism causing the PJI [26, 27] and preoperative synovial fluid characteristics [28] have been investigated and shown to predict two-stage exchange failures (Table 3). Another factor that has been studied is the timing of reimplantation after the first-stage explant is completed. Currently, the results remain inconclusive due to the lack of consistency between past studies in regard to the definition of PJI diagnosis and two-stage exchange failure and perioperative surgical and antibiotic treatment protocols between different institutions. For example, Sabry et al. [12] showed that, in patients with primary TKA PJIs, a longer time between explant and reimplantation was associated with infection recurrence. The reported median duration between the two stages associated with the latter was 103 days (range 2-470 days) whereas Rezaie et al. [29] showed the opposite result. Their analysis determined that the time between the two stages in THA and TKA PJI did not have an impact in predicting two-stage exchange failure.

The literature demonstrates multiple risk factors that lead to two-stage revision failures for THA and TKA including obesity, diabetes mellitus, and immunosuppression [39]. However, not only are these risk factors common for different surgical procedures [40, 41] but they also provide no prognostic value for two-stage revisions for PJI. On the other hand, many oncologic surgical specialties have shown that the novel preoperative scores involving systemic-inflammatory markers are valuable for prognosis [42, 43]. This is likely due to the stimulation of inflammatory and acute-phase proteins in addition to the chemokines, cytokines, and immune cells, all of which contribute to tumor progression and dissemination [44]. In a similar manner, PJI stimulates the inflammatory response and systemic markers and can cause many of the classic clinical symptoms associated with this process such as fevers, erythematous warm joints, and pain [4].

Systemic-inflammatory markers were initially investigated individually as prognostic factors as seen with septic patients. Sepsis, which develops from a local infection that becomes systemic, is an inflammatory process that causes acute organ dysfunction and involves many of the factors mentioned above such as acute-phase proteins, chemokines and cytokines [45]. Simple prognostic factors including interleukin-6 (IL-6) [32] and D-dimer (Table 4) were shown to predict mortality in these patients. Eventually, this led to the development of novel systemic prognostic scores, which encompass a multifactorial presentation of the patient, to predict sepsis mortality [35, 38]. The preoperative systemic-inflammatory based prognosis score, CAR, which predicts 180-day mortality in patients with sepsis [38], was of particular interest because it has successfully predicted prognosis in patients undergoing surgery for soft tissue sarcomas [18], esophageal [19], colorectal [20], and pancreatic cancers [21] and has outperformed other scores including neutrophil/lymphocyte ratio, platelet/lymphocyte ratio, and modified Glasgow Prognostic Score in gastric cancer [46]. Individually, elevated CRP [47] and hypoalbuminemia [39] have been associated with failed two-stage exchange outcomes but the present study showed that the preoperative ratio, which encompasses the inflammatory and nutritional status of a patient, is not a significant indicator of the outcomes of two-stage exchanges after PJI. This is in contrast to the value of CAR as shown in cancer patients.

There were several limitations to this retrospective study. First, the number of patients that met the inclusion criteria was small despite using two different institutional databases. The lack of significant difference of CAR between the successful and failed two-stage exchange was possibly limited by this factor. Secondly, lab values collected up to a year prior to a patient's explant were used to analyze CAR which could not accurately represent the patient's physical state prior to receiving surgery. Finally, patients who had revisions prior to their explant for their two-stage exchange were included, which could also negatively impact the outcomes.

Despite these pitfalls, this study contributes to the literature by applying a successful prognostic model into a field that needs more tools to improve its outcomes. It evaluated the outcomes of two-stage exchanges for THA and TKA in patients from two different institutions who were diagnosed with PJI per the MSIS definition using a novel preoperative systemic-inflammatory prognostic score, CAR. Based on our knowledge of the inflammatory process in PJI and in tumor progression, we hypothesized a correlation, but no significant difference was found with the numbers available. It also highlights the need to prospectively study CAR or other preoperative systemic-inflammation prognostic scores in the setting of PJI as it may provide value in helping to identify the correct timing of reimplantation.

## 5. Conclusion

This study aims to analyze the predictability of two-stage exchange outcomes to treat PJI defined by the MSIS criteria using a novel preoperative systemic-inflammatory score, CAR. Patient data was collected from two institutional databases and CAR did not predict success or failure after a two-stage exchange in the setting of a PJI. Despite the limitations and conclusion, this study provides insight and explores the prognostic possibilities of preoperative inflammatory scores in the setting of PJIs as they have been previously shown to be successful in other surgical field managing diseases driven by inflammation. Further research is needed to explore the application of other scores, such as neutrophil/lymphocyte, in order to better predict a patient's outcome after a two-stage exchange and to alleviate the financial and emotional burden of PJI.

## Figures and Tables

**Table 1:** Demographic and laboratory data of successful and failed two-stage revision cohorts.

	Successful Two-Stage Revision	Failed Two-Stage Revision	p-value
	N = 61	N = 18	
Age (years at primary surgery)	61.0 (56.0, 68.0)	61.0 (54.0, 66.0)	0.578
Gender (female)	28 (45.9)	11 (61.1)	0.257
C-reactive Protein (mg/L)	4.4 (0.8, 9.1)	4.3 (1.8, 10.0)	0.713
Albumin (g/dL)	3.7 (3.1, 4.0)	3.8 (3.5, 4.2)	0.263
CRP/Albumin Ratio (CAR)	1.2 (0.2, 3.0)	1.0 (0.4, 3.2)	0.766

**Table 2:** CAR of successful and failed two-stage revision cohorts within 30-day postoperative period.

	Successful Two-Stage Revision	Failed Two-Stage Revision	p-value
	N = 30	N = 12	
CRP/Albumin Ratio (CAR)	1.20 (0.19, 3.43)	1.03 (0.45, 2.38)	0.945

**Table 3:** Risk factors for two-stage exchange failure and recurrent infections.

Study Authors	Risk Factors
Mortazavi SM, Vegari D, Ho A et al. [26].	Culture-negative PJI; methicillin-resistant PJI; increased reimplantation operative time
Triantafyllopoulos GK, Memtsoudis SG, Zhang W et al. [30].	Female gender, heart and psychiatric diseases
Dwyer MK, Damsgaard C, Wadibia J et al. [28].	Preoperative synovial fluid WBC >60,000 cells/uL, synovial fluid WBC neutrophil >92%, serum ESR >99 mm/hr
Tan TL, Gomez MM, Manrique J et al. [31].	Positive intraoperative culture during reimplantation
Rezaie AA, Goswami K, Shohat N et al. [29].	Charlson comorbidity index
Pelt CE, Grijalva R, Anderson L et al. [14].	(TKA) polymicrobial infection, 4+ surgeries to site of primary TKA before explant
Jhan SW, Lu YD, Lee MS et al. [27].	(THA) BMI 30 kg/m^2^, liver cirrhosis, gram-negative organism, presence of sinus tract
Schwarzkopf R, Mikhael B, Wright E et al. [3].	(THA) Previous history of other prosthetic joint infections

**Table 4:** Prognostic factors for predicting sepsis mortality after two-stage exchange.

Study Authors	Prognostic Factors
Srisangthong P, Wongsa A, Kittiworawitkul P and Wattanathum A [32].	Plasma IL-6 100 pg/mL (28-day mortality)

Parlato M, Cavaillon JM [33].	Lactate, pentraxin 3 (PTX3), pancreatic stone protein, IL-8; microRNA markers 15a/16/193/483-5p, CD14 and CD64 (28-day mortality)

Rodelo JR, De la Rosa G, Valencia ML et al. [34].	D-dimer (28-day mortality)

Artero A, Zaragoza R, Zamarena JJ et al. [35].	Hypoalbuminemia

Chen Y, Chunsheng L [36].	Plasma brain natriuretic peptide >113 pg/mL (28-day mortality)

Ranzani OT, Zampieri FG, Forte DN et al. [37]; Kim MH, Ahn JY, Song JE et al. [38].	CAR (90-day [37] and 180-day [38] mortality)

## Data Availability

The patient data used to support the findings of this study are restricted by the local Institutional Review Board in order to protect patient privacy.
